# Mössbauer, optical, magnetic, electrochemical and antibacterial studies of the Ag/Sr_0.85_Ag_0.15_FeO_3−δ_ composite

**DOI:** 10.1038/s41598-025-07635-w

**Published:** 2025-07-03

**Authors:** E. K. Abdel-Khalek, Mamduh J. Aljaafreh, Ahmad Saleh, M. M. Osman

**Affiliations:** 1 Department of Physics, Faculty of Science, Al-Azhar University, Nasr City11884, Cairo, Egypt; 2 Physics Department, College of Science, Imam Mohammad Ibn Saud Islamic University (IMSIU), Riyadh, 11623 Saudi Arabia; 3 Department of Mathematics and Natural Sciences, Prince Mohammad Bin Fahd University, Al Khobar, 31952 Saudi Arabia

**Keywords:** Composite, Sol-gel, Mössbauer, Exchange bias, Electrochemical, Antibacterial properties, Energy science and technology, Materials science

## Abstract

In this study, Ag/Sr_0.85_Ag_0.15_FeO_3−δ_ composite was prepared by sol-gel method. The structure, morphology, and surface properties of this composite have been characterized by X-ray diffraction (XRD) combined with Rietveld analysis, transmission electron microscopy (TEM), X-ray photoelectron spectroscopy (XPS), and Mössbauer spectroscopy. XRD and TEM results confirm the existence of the perovskite phase of Sr_0.85_Ag_0.15_FeO_3−δ_ and Ag nanoparticles. XPS and Mössbauer spectroscopy results indicate the existence of oxygen vacancies (δ) and different oxidation states of Fe ions. The band gap energy (E_g_) of this composite is 3.53 eV and lies in the UV region. The magnetic hysteresis loop (M-H) of this composite reveals the existence of an exchange bias effect because of the competition between antiferromagnetic (AFM) and ferromagnetic order (FM). Electrochemical measurements indicated that the Ag/Sr_0.85_Ag_0.15_FeO_3−δ_ composite exhibits pseudocapacitive behavior. The present composite showed strong antimicrobial activity to fight both tested Gram-positive and Gram-negative bacteria as well as unicellular and filamentous fungi strains.

## Introduction

Recently, Ag doped SrFeO_3−δ_ perovskites have attracted attention because of its excellent electronic, optical, magnetic, electrochemical, antibacterial properties which due to the existence of Ag^+^, oxygen vacancies (δ), and mixed valence states of Fe ions^1–3^. Consequently, they have various applications such as fuel cells, catalysts, electrochemical sensors, supercapacitors, and antibacterial agents for the types of bacteria that resist the antibiotic^4,5^. The crystal structure of SrFeO_3−δ_ perovskite depends on the concentration of oxygen vacancies values (δ)^4,6^. Therefore, below δ = 0.25, the SrFeO_3−δ_ perovskite was crystallized in an orthorhombic structure which is composed of Fe^4+^O_5_ square pyramidal and Fe^3+^O_6_ distorted octahedra^4,6^. The valence states, spin state, and the coordination of Fe as well as oxygen vacancies content in SrFeO_3−δ_ perovskite can be determined by the Mössbauer spectroscopy technique^7,8^. Abd El-Naser et al.^8^ have analyzed the Mössbauer spectrum of SrFeO_3−δ_ perovskite and they found that there is a magnetic sextet, two quadrupole doublets, and a single line. They also found that the oxygen vacancies content is 0.135. Rosa et al.^2^ investigated the magnetic and electronic properties of Ag-doped SrFeO_3_ perovskite using quantum simulations and demonstrated that Ag-doping leads to the modification of the magnetic order through the change in the concentration of oxygen vacancies (δ). Williams et al.^4^ investigated the magnetic properties of Sr_4_Fe_4_O_11_ orthorhombic phase and they found that Sr_4_Fe_4_O_11_ orthorhombic exhibits an exchange bias effect near Néel temperature (T_N_ ~230 K).

In modern experiments, SrFeO_3−δ_ perovskite and its derivatives have been investigated as a promising electrode material for energy storage applications^9,10^. Further, Ag-doped perovskite oxides lead to the improvement of specific capacitance and cyclic stability because of Ag-comprising electrode has high activity in the reaction of oxygen reduction^11,12^. Lang et al.^12^ studied the Ag nanoparticles decorated La_0.85_Sr_0.15_MnO_3_ as electrode materials of supercapacitors and demonstrated that their presence leads to the acceleration of charge transfer and contributes in pseudocapacitance with a small amount. Ahangari et al.^9^ studied the SrFeO_3_ and SrCo_0.5_Fe_0.5_O_3_ perovskites as electrode materials of supercapacitors and found that the partial substitution of Fe by Co leads to the reduction in the specific capacitance.

Ag nanoparticles in nanocomposite are safe potent antibacterial agent and nontoxic as well as they are used for killing much types of bacteria that cause diseases^5,13^. Abdel-Khalek et al.^3,7^ studied the antibacterial activities of Ag-doped SrFeO_3−δ_ perovskite. They found that the addition of a low concentration of Ag (0.05 and 0.10) leads to the enhancement of its antibacterial activity owing to inherent bacteria resistance for Ag nanoparticles. Duran et al.^13^ compared the antibacterial activities between the silver ions (Ag^+^) and silver nanoparticles. They found that the Ag^+^ ions lead to inactivation of bacteria because they react with proteins in the thiol groups^14^. Li et al.^15^ discussed the antibacterial activities mechanism of silver nanoparticles through three possible mechanisms. First, silver nanoparticles can be directly attached to the cell membrane and consequently damage it. Second, silver nanoparticles can be penetrated inside the bacteria and consequently lead to DNA damage^13^. Third, silver nanoparticles can generate the reactive oxygen species and consequently lead to the death of bacteria^16^.

In view of the aforementioned aspects, the significant problem of SrFeO_3−δ_ perovskite as an electrode material is its low performance and the unstability of its structure^11,17^. In order to solve this problem, we fabricate the Ag/Sr_0.85_Ag_0.15_FeO_3−δ_ composite by sol-gel method. This composite contains mixed electronic (Fe^3+^/Fe4^+^) and ionic (Ag) conductors which are able to transfer both ions and electrons at the same time. In addition, we expect the presence of both the silver ions (Ag^+^) and silver nanoparticles in the Ag/Sr_0.85_Ag_0.15_FeO_3−δ_ composite which enhancement the antibacterial activity of this composite. Therefore, the aim of the present work is the improvement of the electrochemical and antibacterial properties of Ag/Sr_0.85_Ag_0.15_FeO_3−δ_ composite as electrode materials and antibacterial agent.

## Experimental

### Sample preparation

**Fig. 1:**
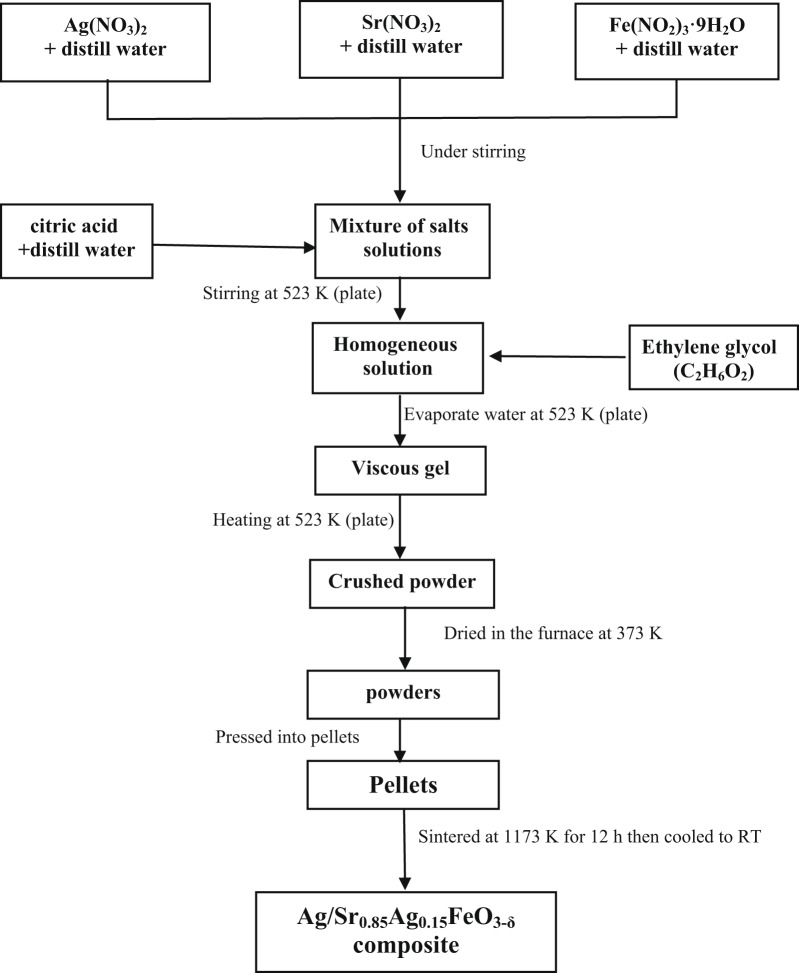
Flowchart of the sol-gel method for the synthesis of the Ag/Sr_0.85_Ag_0.15_FeO_3−δ_ composite.

Ag/Sr_0.85_Ag_0.15_FeO_3-δ_ composite was prepared by sol-gel method using citric acid (C_6_H_8_O_7_) as a complexing agent. The grade chemicals Sr(NO_3_)_2_ (99%) and Fe(NO_2_)_3_·9H_2_O (98%) were obtained from Loba Chemie while Ag(NO_3_)_2_ (99%), citric acid C_6_H_8_O_7_ (99.8%) and ethylene glycol C_2_H_6_O_2_ (99.5%) were obtained from Alpha Chemika. The experimental details about the preparation method of this composite were reported in a previous work^3^. The experimental flowchart of the sol-gel method for the preparation of Ag/Sr_0.85_Ag_0.15_FeO_3-δ_ composite is shown in Fig. 1.

### Characterization

X-ray diffraction (XRD) pattern of this composite at room temperature was carried out by a Bruker Co D8 Discover X-ray diffractometer with a Cu Kα radiation source (λ = 1.5406 Å) in the 2θ range of 10° − 80° and scanning rate of 0.02°. Rietveld analysis of XRD data of this composite was performed by Fullprof suite. The transmission electron microscopy (TEM) image and selected area electron diffraction (SAED) pattern were obtained using a JEM-2100 PLUS (Japan) electron microscope. The used accelerating voltage is 200 kV. The energy dispersive X-ray spectroscopy (EDS) elemental mapping of this composite was determined using a JEM-2100 F (URP) instrument. X-ray photoelectron spectroscopy (XPS) measurement of this composite was performed by K-Alpha^™^ photoelectron spectrometer (Thermo Scientific^™^ (USA)) using a monochromatic AL-K_α_ radiation source. The Mössbauer spectrum of this composite was measured by a conventional constant acceleration spectrometer with ^57^Co (embedded in a Cr matrix) radioactive source at room temperature. The absorption spectrum of this composite was carried out using UV-Vis. spectrophotometer (Jasco-V-570) in the wavelength range from 200 to 800 nm. The room temperature magnetization hysteresis (M-H) loop of this composite was performed by vibrating the sample magnetometer (VSM of Lake Shore 7410).

### Electrochemical properties study

The electrochemical properties of the composite were performed in a 6 M KOH aqueous electrolyte using a potentiostat-galvanostat unit (OrigaFlex, France). The electrochemical measurements were studied in three electrode cells using a nickel foam coated with the present composite as a working electrode, platinum (Pt) plate as the counter electrode, and Hg/HgO as the reference electrode. The cyclic voltammetry (CV) measurements of this composite were performed within a potential window of 0 to 0.5 V at different scanning rates (5, 10, 25, 50, and 100 mV/s). The galvanostatic charge/discharge (GCD) measurements of this composite were performed at different current densities (1, 2, 3, 4, and 5 A/g) over a potential window of 0 to 0.5 V. The electrochemical impedance spectroscopy (EIS) measurement of this composite was performed over the frequency range of 100 kHz to 100 MHz using Nyquist plot. The specific capacity (C) of this composite was estimated through the GCD curves by the following Eq. 

1 $$C=\frac{{I \times dt}}{m}(C/g)$$

where I is the discharging current (A), dt represents the discharge time, and m is the mass of active material (g) within the electrode. Further, the specific capacitance (C_s_) of this composite was estimated through the GCD curves by the following relation.

2 $${C_s}=\frac{{2I\smallint Vdt}}{{m{{(\Delta V)}^2}}}\quad (F/g)$$

where ∫Vdt represents the area under the discharge of the GCD curve, and ΔV is the discharging potential range (V).

### Antibacterial properties study

The antimicrobial activity in vitro of the Ag/Sr_0.85_Ag_0.15_FeO_3−δ_ composite in different concentrations against a wide range of pathogens was studied using the agar disc diffusion method^3^. Gram-positive (*Bacillus spizizenii* (ATCC 6633) and *Staphylococcus aureus* (ATCC 6538)) and Gram-negative (*Pseudomonas aeruginosa* (ATCC 9027) and *Escherichia coli* (ATCC 8739)) bacteria were studied against the investigated composite. Further, unicellular (*Candida albicans* (ATCC 10231)) and filamentous (*Aspergillus brasiliensis (ATCC 16404))* fungi were studied against the investigated composite. Standard antibacterial and antifungal agents were Imipenem (IPM 10) and Fungzol, respectively. In accordance CLSI guidelines^18^, the inhibition zones diameters were determined after 24 h of incubation for bacteria and 3 days for fungi at 37 °C.

## Results and discussion

### XRD, TEM, SAED, and HRTEM studies

**Fig. 2:**
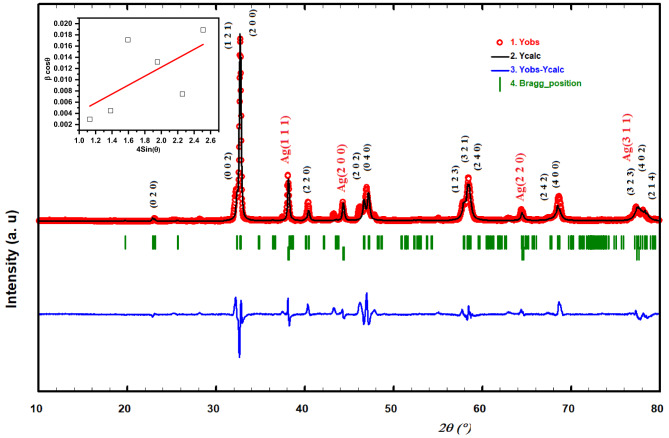
Rietveld refinement XRD patterns of the present composite and the diffraction peaks with stars are attributed to the metallic Ag as well as the inset displays the plot of βcosθ against 4sinθ for Sr_0.85_Ag_0.15_FeO_3−δ_ perovskite.

Figure 2 displays the Rietveld refined XRD pattern of the Ag/Sr_0.85_Ag_0.15_FeO_3−δ_ composite. This pattern showed the coexistence of orthorhombic (space group Pnma) and cubic (space group Fm3m) phases for Sr_0.85_Ag_0.15_FeO_3−δ_ perovskite and Ag metallic, respectively^19–22^. The Rietveld refinement parameters of this composite such as the phase fraction, lattice parameters, unit cell volume, atoms, Wyckoff position, lattice coordinate, and reliability R-factors (R_B_ and R_F_) are tabulated in Table 1. From this table, it is noticed that the crystal structure of the present composite consists of dominant orthorhombic phase (81.53%) for Sr_0.85_Ag_0.15_FeO_3−δ_ perovskite with a minor cubic phase (18.47%) for Ag metallic. By comparing the obtained XRD result with the previous results for Sr_1 − x_Ag_x_FeO_3−δ_ (where x = 0.05 and 0.10) perovskite, the transition from cubic (Pm-3 m) to orthorhombic structure (Pnma) was occurred with increasing Sr content to x = 0.15^3^. This transition can be attributed to the presence of high amount of oxygen vacancies as confirmed by the following XPS and Mössbauer results^23^. The crystallite size (D) of Sr_0.85_Ag_0.15_FeO_3−δ_ perovskite has been estimated by the following Williamson-Hall model^24^.

3 $$\beta {\text{cos}}\theta ={\text{4}}\varepsilon {\text{sin}}\theta +({\text{k}}\lambda /{\text{D}})$$

where β is the difference in full-width at half maximum (FWHM) between the observed broadening (β_obs_) of the XRD peaks of perovskite in radiant and the instrumental broadening (β_std_)^25^. The β_std_ value was estimated from the XRD pattern of standard Corundum Al_2_O_3_ sample. Additionally, ε is the microstrain, θ is the diffraction angle of the peaks, k = 0.9 is the constant, and λ = 0.15406 nm is the wavelength of the X-ray radiation source. The inset of Fig. 2 displays the plot of βcosθ against 4sinθ for Sr_0.85_Ag_0.15_FeO_3−δ_ perovskite. The D value of the Sr_0.85_Ag_0.15_FeO_3−δ_ perovskite can be estimated from the y-intercept (kλ/D) of the fitted line and it is equal to 37.47 nm^26^.

**Table 1:** TRietveld refinement results of Ag/Sr_0.85_Ag_0.15_FeO_3-δ_ composite.

Crystal structure	Lattice parameters	Atom	Wyckoff position	Lattice coordinates			Reliability factor
Orthorhombic (Pnma)	a=5.4734 Å	Sr/Ag	4c	0.506	0.250	0.002	R_B_=12.5
(81.53%)	b=7.7068 Å	Fe	4b	0.000	0.000	0.000	R_F_= 9.10
	c=5.5369 Å	O1	8d	–0.008	0.250	–0.131	
	V= 233.5575 Å^3^	O2	4c	0.252	0.023	0.337	
Cubic (Fm3m)	a=b=c= 4.0859 Å	Ag	4a	0.000	0.000	0.000	R_B_=10.20
(18.47%)	V= 68.2137 Å^3^						R_F_= 6.30

**Table 2:** The Mössbauer parameters of the Ag/Sr_0.85_Ag_0.15_FeO_3−δ_ composite.

Valence state of iron ions	IS (mm/s)	QS/ε (mm/s)	B (T)	A %
Magnetic Fe	0.406	–0.765	39.7	55.4
Fe^4+^	–0.057	0.261	–	22.5
Fe^3+^	0.251	0.548	–	22.1

IS, QS/ε, B and A are the isomer shift relative to metallic iron at RT, the quadrupole splitting of a doublet in the paramagnetic state/ the quadrupole shift, the average magnetic hyperfine field of magnetic sextet, and the relative area, respectively.

**Table 3:** Antimicrobial activity assessment of the Ag/Sr_0.85_Ag_0.15_FeO_3−δ_ composite.

Classification	Test strains	Inhibition zone diameter (mm) of present sample at concentrations (mg)			
		5	10	20	Imipenem/ Fungzol Standards
Gram positive bacteria	*Bacillus spizizenii* ATCC6633	7	9	10	25
	*Staphylococcus aureus* ATCC 6538	10	15	22	0.0 (R)
Gram negative bacteria	*Pseudomonas aeruginosa* ATCC 9027	9	12	16	0.0 (R)
	*Escherichia coli* ATCC 8739	7	8	9	22
Unicellular fungi	*Candida albicans* ATCC 10,231	9	11	14	35
Filamentous fungi	*Aspergillus brasiliensis ATCC 16,404*	0	0	30	21

**Fig. 3:**
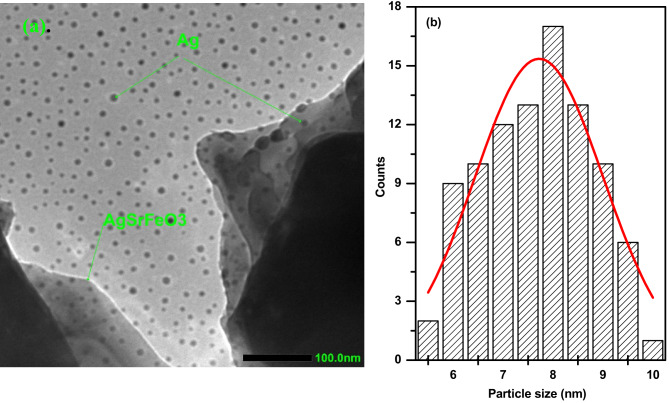
**a** and **b** The transmission electron microscopy image of present composite and the particle size distribution histogram of Ag^+^ particles, respectively.

**Fig. 4:**
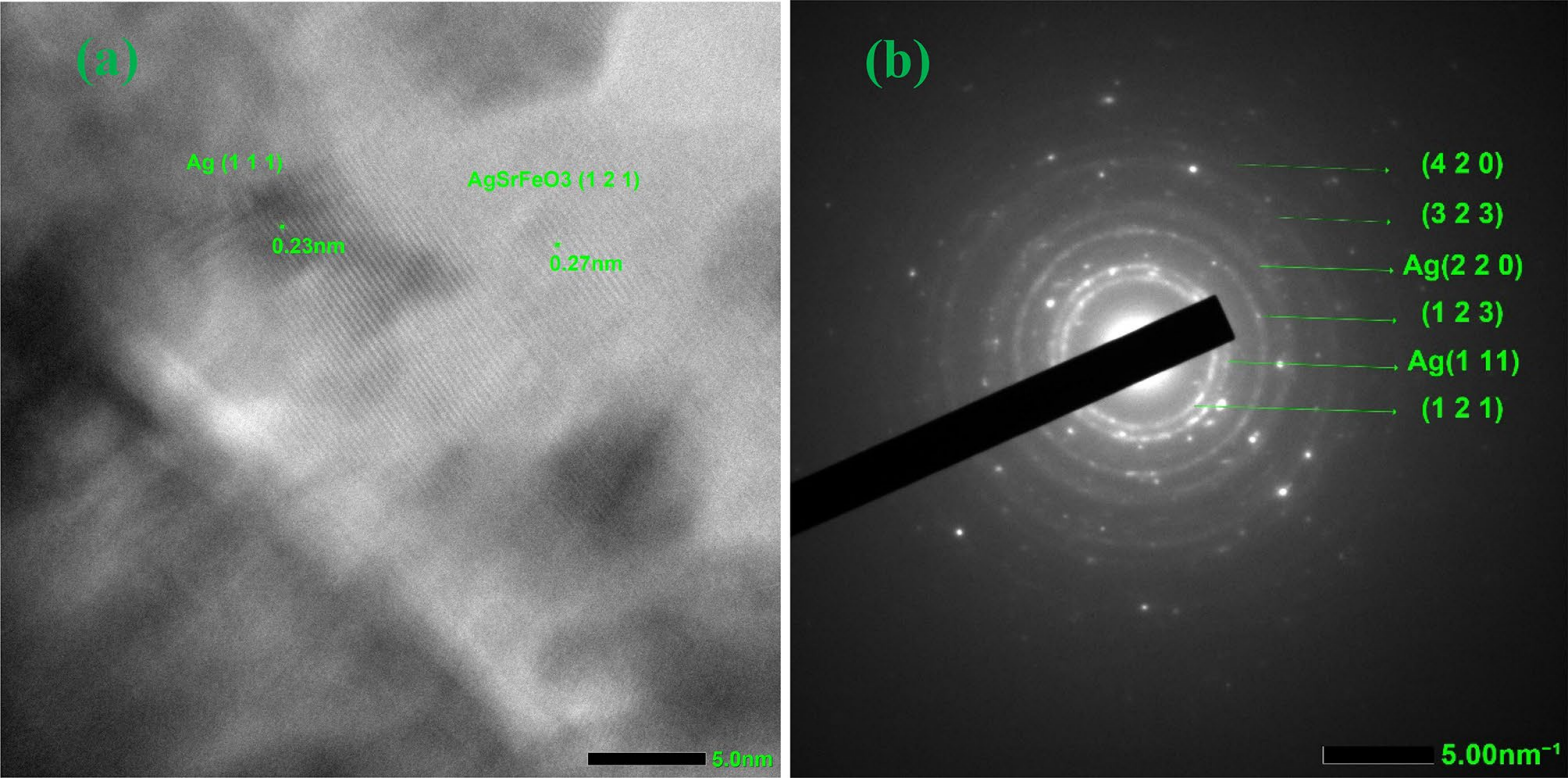
**a** and **b** The high-resolution TEM (HR-TEM) image of the present composite and the selected area electron diffraction (SAED) pattern, respectively.

**Fig. 5:**
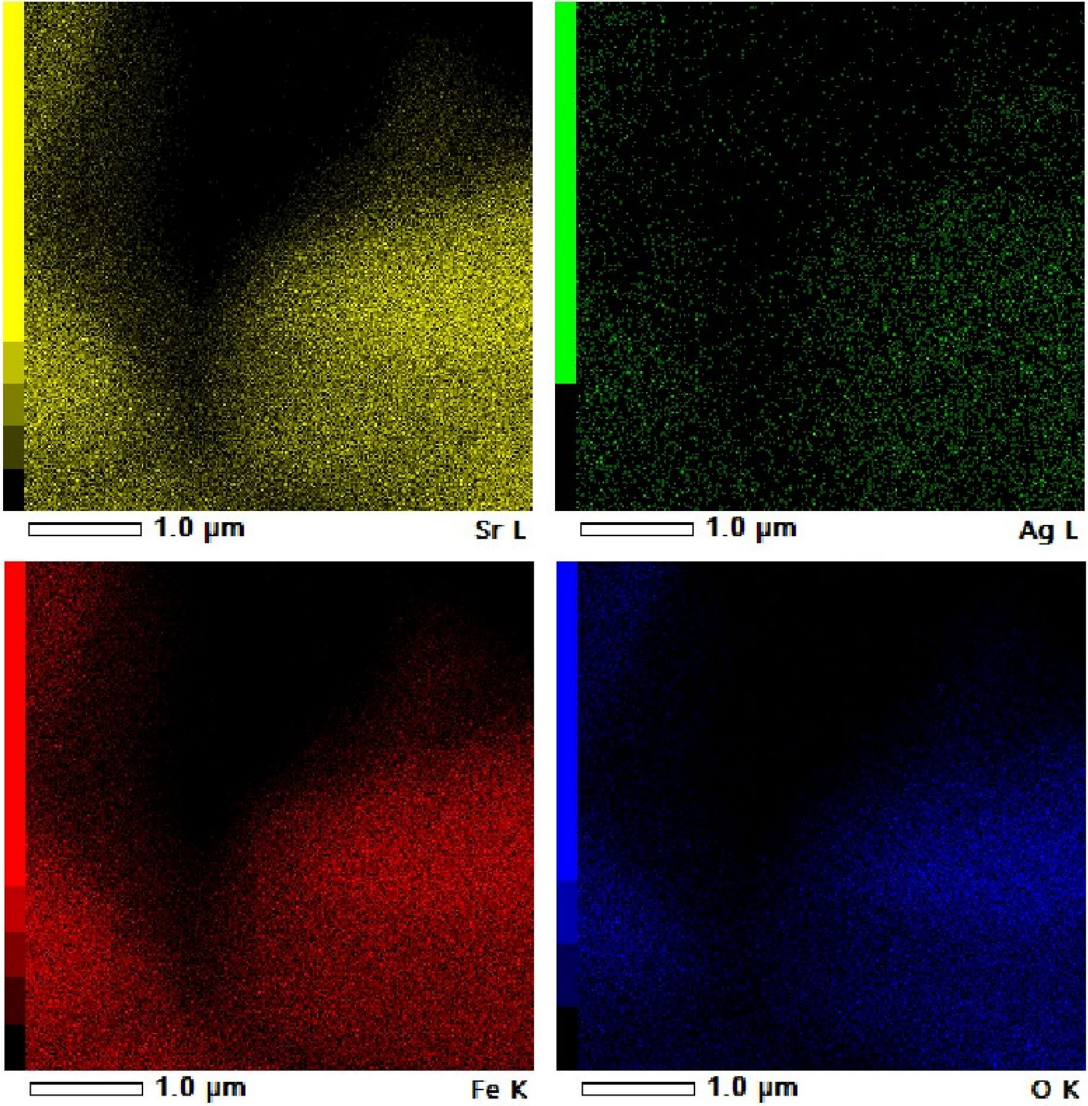
The energy dispersive X-ray spectroscopy (EDS) mapping images of the present composite.

Figure 3a and b displays the TEM image of the Ag/Sr_0.85_Ag_0.15_FeO_3-δ_ composite and the particle size distribution histogram of Ag particles, respectively. The image revealed the presence of aggregated sheets of the Sr_0.85_Ag_0.15_FeO_3-δ_ perovskite and contains small spherical particles of Ag^1,3^. This observation provides evidence for the presence of Sr_0.85_Ag_0.15_FeO_3-δ_ perovskite and metallic Ag in the present composite, which is in agreement with the above XRD results. The aggregation of the Sr_0.85_Ag_0.15-x_FeO_3-δ_ perovskite in this composite can be attributed to their magnetic properties, in agreement with the following magnetic results^27^. The histogram of Ag particles, fitted using a Gaussian function, exhibits a narrow particle size distribution as shown in Fig. 3b which indicates the particle size distribution is uniformed. The estimated average particle size of Ag from the histogram is 7.72 nm. Figure 4a and b displays the HRTEM image and SAED pattern of the Ag/Sr_0.85_Ag_0.15_FeO_3-δ_ composite, respectively. The HRTEM image exhibits two types of lattice fringes which can be attributed to Sr_0.85_Ag_0.15_FeO_3-δ_ perovskite and Ag nanoparticle, proving the presence of them in this composite^3^. This result is in agreement with previous result for Ag/SrFeO_3_ composite^1^. The observed interplanar distance values of 0.27 and 0.23 nm indexes to the d spacings of the (1 2 1) and (1 1 1) crystal planes of the orthorhombic phase of Sr_0.85_Ag_0.15_FeO_3-δ_ perovskite and the cubic phase of metallic Ag, respectively^1^. This finding agrees with the d-values which obtained from the XRD analysis for the Sr_0.85_Ag_0.15_FeO_3-δ_ perovskite and matched with the JCPDS no. 87–0718 for Ag^21^. From Fig. 4b, SAED pattern exhibits diffraction rings with relatively sharp spots which indicate the polycrystalline nature of this composite^7,8^. The planes of (1 2 1), (1 2 3), (3 2 3) and (4 2 0) are assigned to orthorhombic structure of Sr_0.85_Ag_0.15_FeO_3-δ_ perovskite while the others planes of (111) and (220) planes are assigned to a cubic structure of metallic Ag^28^. This result is in agreement with the previous result for Ag/SrFeO_3_ composite^1^. Figure 5 displays the EDS mapping images of the Ag/Sr_0.85_Ag_0.15_FeO_3-δ_ composite. The EDS mapping images indicate the existence of Sr (yellow), Ag (green), Fe (red), and O (blue) elements without any impurities. Moreover, the distribution of Ag element in metallic Ag was observed in different regions while the distribution of Ag, Sr, Fe, and O elements in Sr_0.85_Ag_0.15_FeO_3-δ_ perovskite are uniform, consistent with the XRD and TEM analysis.

### XPS and Mössbauer studies

**Fig. 6:**
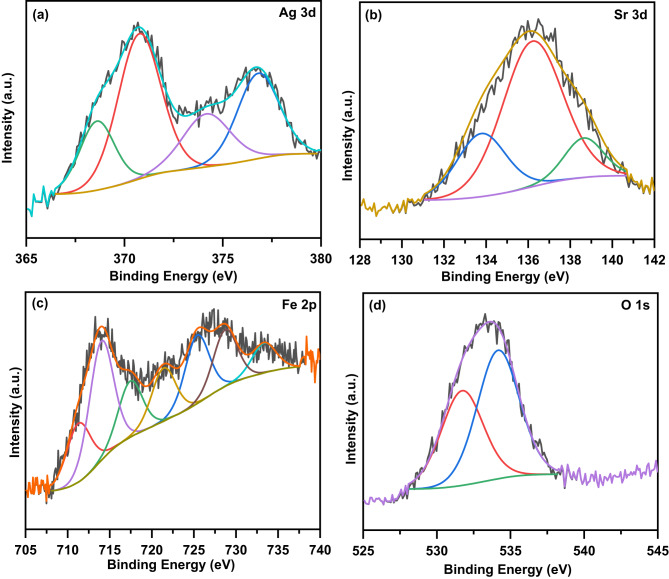
**a**, **b**, **c**, and **d** Deconvoluted XPS spectra of Ag 3d, Sr 3d, Fe 2p, and O 1s of the present composite, respectively.

Figure 6a, b, c, and d displays the deconvoluted XPS spectra of Ag 3d, Sr 3d, Fe 2p, and O 1s regions of the composite, respectively. It is notice that the XPS spectra in this composite indicated the existence of Ag, Sr, Fe, and O elements without any impurities, in agreement with the EDS mapping results. According to Fig. 6a, the XPS spectrum of the Ag 3d was deconvoluted into four peaks at 368.6, 370.76, 374.17, and 376.81 eV^29,30^. The double peaks at 368.6 and 374.17 eV are assigned to the metallic Ag 3d_5/2_ and Ag 3d_3/2_ states, respectively^30,31^. These peaks can be attributed to the reduction of Ag in AgNO_3_ during sol-gel process; thus, some Ag species exist on the surface of the perovskite sheets^29,32^. Additionally, the double peaks at 370.76 and 376.81 eV are assigned to the Ag^1+^ 3d_5/2_ and Ag^1+^ 3d_3/2_ states, which can be attributed to the oxidation of some Ag species to Ag_2_O after sintering in air^29^. According to Fig. 6b, the XPS spectrum of Sr 3d was deconvoluted into three peaks at 133.77, 136.36, and 138.73 eV. The peak at 136.36 eV is assigned to the 3d_3/2_ of Sr^2+^ ions into the perovskite structure^7,27^. The two peaks at 133.77 and 138.73 could be ascribed to 3d_5/2_ of Sr^2+^ ions accompanied with oxygen vacancies^27,33^. According to Fig. 6c, the XPS spectrum of Fe 2p was deconvoluted into six peaks of 2p_3/2_ and 2p_1/2_ electron levels and a weak satellite peak. These peaks are at 711.29, 714, 717.44, 721.4, 725.38, 728.72, and 732.34 eV. The double peaks at 711.29 and 725.38 eV are assigned to 2p_3/2_ and 2p_1/2_ states of Fe^3+^, respectively^29,34^. Further, the double peaks at 714 and 728.72 eV are due to 2p_3/2_ and 2p_1/2_ states of Fe^4+^ ions, respectively^2^. The satellite peak at 717.44 eV could be ascribed to 2p_3/2_ of Fe^3+^ accompanied by oxygen vacancies^7,29^. The additional sub-peak at 721.4 eV could be ascribed to 2p_1/2_ of Fe^3+^ ions while the peak at 732.34 eV is due to Fe 2p_1/2_ and can be attributed to the satellite structure^7,29^. From the above observation, it can be concluded that the presence of mixed oxidation states of Fe ions (3 + and 4+). According to Fig. 6d, the XPS spectrum of O 1s was deconvoluted into two peaks at 531.72 and 534.17 eV. This result showed that there are two kinds of oxygen states at the surface of this composite^8,29^. The peak at 531.72 eV is corresponding to the lattice oxygen (O_latt_) which is due to the contribution of Ag-O, Sr-O, and Fe-O in the crystal lattice^29,30^. In addition, the peak at 534.17 eV is assigned to the surface adsorbed oxygen (O_ads_) which resulted from oxygen vacancies^8,35^. The percentages of O_latt_ and O_ads_ are 42.39 and 57.61, respectively, which can be obtained from the fitting of XPS spectrum as shown in Fig. 6d. Therefore, the ratio of O_ads_/O_latt_ is 1.36 which means that the present composite has a high content of oxygen vacancy^17^.

**Fig. 7:**
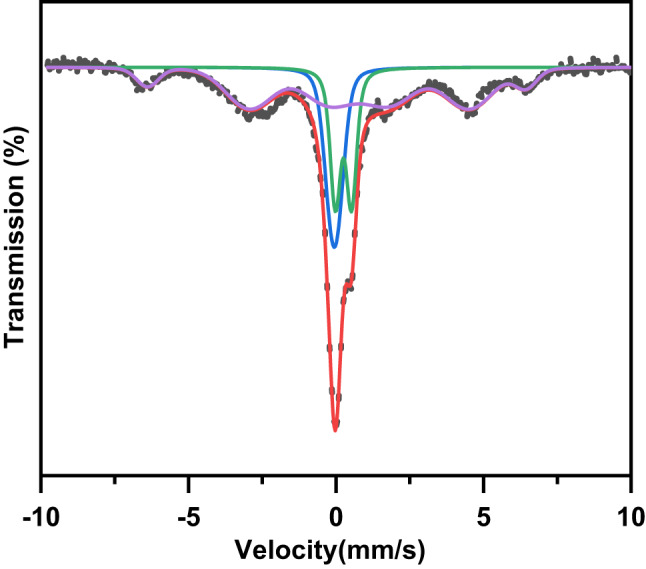
The Mössbauer spectrum of Ag/Sr_0.85_Ag_0.15_FeO_3−δ_ composite recorded at room temperature.

Figure 7 displays the Mössbauer spectrum at room-temperature for the present composite. It is noticed that this spectrum is composed of magnetic sextet, single line, and doublet. The appearance of magnetic sextet in this spectrum indicated the existence of magnetic order of AFM while the appearance of paramagnetic doublet indicated the existence of small amount ferromagnetic particles (superparamagnetic)^36–39^. This observation is supported by the following magnetic results and agreement with the previous report for SrFeO_3−δ_ ceramic^8^. Further, the single line and paramagnetic doublet in this spectrum indicate presence of Fe in two different valence states^7,8^. The Mössbauer parameters such as isomer shift (IS), quadrupole splitting/quadrupole shift (QS/ε), magnetic field (B), and the relative area (A) are listed in Table 2. The magnetic sextet has a value of IS = 0.406 mms^− 1^ and a magnetic field of 39.7 T with small ε = -0.765 mms^− 1^ which is due to Fe^3+^ ions in the octahedral site^40–42^. The single line has a low value of IS = − 0.057 mms^− 1^ with QS = 0.261 mms^− 1^ which is due to Fe^4+^ ions in octahedral coordination^38,42^. The doublet has IS = 0.18 mms^− 1^ with QS = 0.548 mms^− 1^ which is due to Fe^3+^ ions in six coordinated octahedrons^44^. This observation indicates the existence of the orthorhombic phase that was detected by the above XRD result^42^. From Table 2, the QS value of Fe^3+^ is larger than that of Fe^4+^ which resulted from the presence of oxygen vacancies with Fe^3+^ ions^43^. Based on the charge balance between Fe^4+^ and Fe^3+^ ions, the oxygen vacancies parameter (δ) in this composite can be estimated by using the following relation^7^.

4 $$(3 - \delta )=25+0.5{f_{F{e^{4+}}}}$$

where ${f_{F{e^{4+}}}}$ represent the fractions of Fe^4+^ ions in the sample. The δ value in this composite is 0.38, indicating the presence of orthorhombic phase of Sr_0.85_Ag_0.15_FeO_3-δ_ perovskite.

### Optical, magnetic, and electrochemical studies

**Fig. 8:**
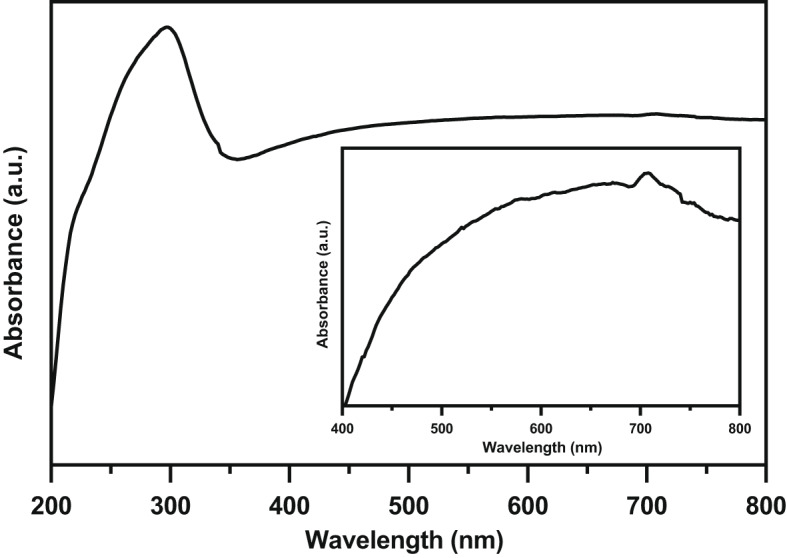
The UV-Vis. absorption spectrum of the Ag/Sr_0.85_Ag_0.15_FeO_3−δ_ composite in the wavelength range of 200–800 nm and the inset shows the magnified the Vis. range.

**Fig. 9:**
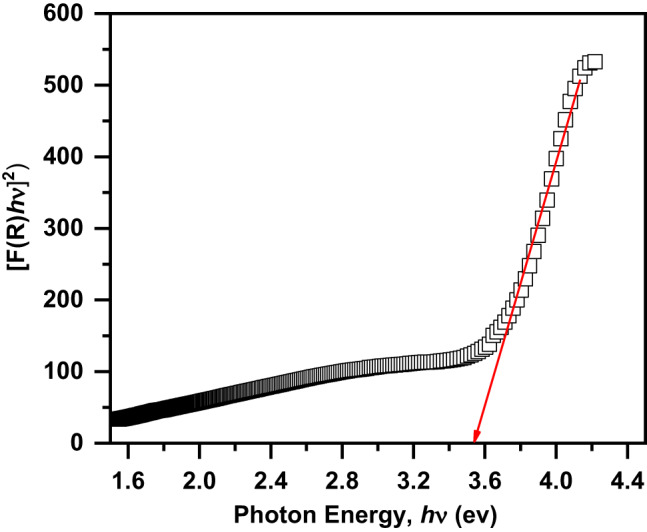
The plot of (F(R) hν)^2^ against hν of the Ag/Sr_0.85_Ag_0.15_FeO_3−δ_ composite.

Figure 8 displays the UV-Vis. absorption spectrum of the present composite. In the UV range, the spectrum exhibits absorption peak at 298 nm while in the Vis. range, the spectrum exhibits two board absorption peaks around 580 and 708 nm (as shown in the inset of Fig. 8). The absorption peak in the UV range may be due to the transfer of charge from O 2p level to 3e_g_ orbital^3,7^. Additionally, the absorption peak in Vis range may be due to the transfer of charge from O 2p to Fe 3d accompanied with oxygen vacancies^3,7^. The band gap energy (E_g_) of the present composite can be estimated from Wood and Tauc’s equation^45^.

5 $$\alpha (hv)=C{(hv - {E_g})^n}$$

where α is the absorption coefficient, hν is the photon energy, C is a constant, and n is the exponent depends on the nature of the transition (n = ½ for a direct allowed transition)^3^. The absorption coefficient (α) can be calculated from Kubelka-Munk (K-M) function F(R) by the following Eqs.^7,46^.

6 $$\alpha \approx F(R)={(1 - R)^2}/(2R)$$

where R is the diffuse reflectance. Figure 9 displays the plot of (F(R) hν)^2^ against hν for the present composite. The E_g_ value of the present composite can be estimated from the intersection of tangent line with (F(R) hν)^2^=0. The E_g_ value of this composite is 3.53 eV that lies in the UV region. This value indicates that the present composite has semiconductor properties^27,47^. Additionally, the small E_g_ value is due to the existence of oxygen vacancies close to the conduction band which cause the formation of extra energy levels^45^.

**Fig. 10:**
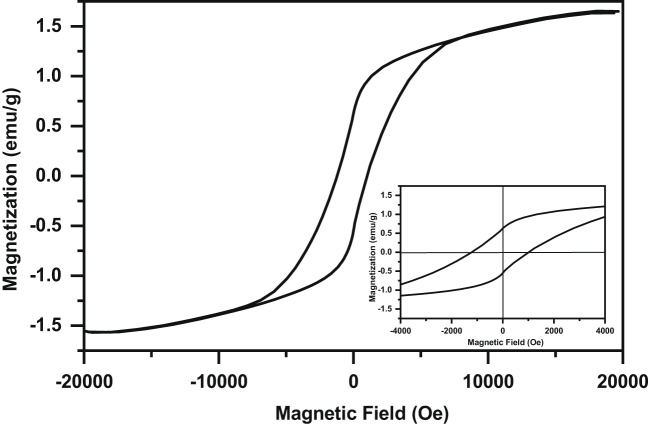
The M-H hysteresis loop of the Ag/Sr_0.85_Ag_0.15_FeO_3−δ_ composite at room temperature.

Figure 10 displays the room temperature M-H hysteresis loop of the present composite. This loop shows the presence of mainly AFM with small fraction of ferromagnetic order^3,27^. This finding is in agreement with above Mössbauer result and is similar to that reported by Abdel-Khalek et al. for Sr_1-x_Ag_x_FeO_3-δ_ (where x = 0.05 and 0.10) perovskite sample^3^. The AFM order in this composite may be due to the superexchange (SE) interaction of Fe^3+^−O − Fe^3+^ and Fe^4+^−O − Fe^4+^^3,48^. The small fraction of ferromagnetic (FM) order in this composite is due to the double exchange (DE) interaction between Fe^4+^−O − Fe^3+^^3,49^. The magnified of the central region of the M-H loop of this sample is shown in the inset of Fig. 10. It is noticed that the M-H loop is asymmetric and displays a shift in both the negative field and positive magnetization axes^4,50^. This observation shows the presence of an exchange bias phenomenon in the present composite which is similar to that reported by Williams et al. and Kumar et al. for SrFeO_x_ and Ti doped SrFeO_3-δ_, respectively^4,50^. The exchange bias effect in this composite is due to the exchange coupling between the AFM ordered moments and FM-ordered spins at the interface between them^4,50^. The existence of the exchange bias phenomenon at room temperature in this composite makes it a promising material for magnetic applications^4,48^. The magnetic parameters, magnetization at maximum applied field (M_s_), remanent magnetization (M_r_), and coercive field (H_c_) of this composite are 1.6100 emu/g, 0.58877 emu/g, and 1117.7 G, respectively. The smaller value of M_r_ can be attributed to the coexistence of FM within AFM^26^. The magnetic parameters for this composite are greater than that reported by Abdel-Khalek et al. for the Sr_0.90_Ag_0.10_FeO_3-δ_ perovskite sample which may be due to the increase in oxygen vacancies as discussed earlier in XPS and Mössbauer results^3^. It is known that the value of the squareness parameter (k = M_r_/M_s_) indicates the type of interaction between nanoparticles. The k value for the present composite is 0.365 which indicates that the type of interaction between nanoparticles is magnetostatic^51^. Further, the large value of H_c_ in this composite indicates the transforms of the hysteresis loop from soft to hard^3,51^.

**Fig. 11:**
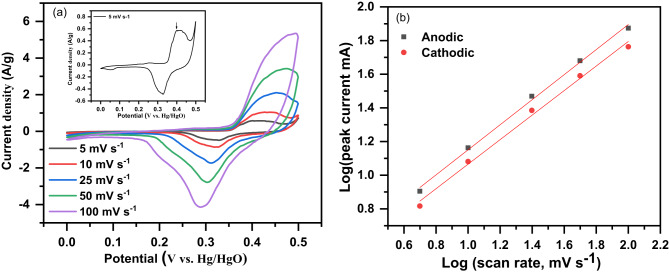
**a** and **b** The cyclic voltammetry (CV) curves of the Ag/Sr_0.85_Ag_0.15_FeO_3−δ_ composite at different scanning rates of 5, 10, 25, 50, and 100 mVs^− 1^ and the plot of log (i) against log (ν) for the present composite, respectively.

Figure 11a displays the CV curves of the Ag/Sr_0.85_Ag_0.15_FeO_3−δ_ electrode at different scan rates of 5, 10, 25, 50, and 100 mV/s. The CV curves exhibit redox peaks which indicate that the present electrode exhibits typical pseudocapacitive behavior^1,9^. The presence of redox peaks in the present electrode can be attributed to the Fe^3+^/Fe^4+^ and Ag/Ag_2_O redox reactions in the electrolyte^9,12^. The small redox peaks of Ag/Ag_2_O in this composite (as shown in inset Fig. 11a) are similar to that reported by Lang et al. and Lang et al. for Ag/La_0.85_Sr_0.15_MnO_3_ and Ag/La_0.7_Sr_0.3_CoO_3−δ_ perovskite sample^12,22^. In addition, the presence of δ in the Ag/Sr_0.85_Ag_0.15_FeO_3−δ_ composite leads to the enhancement of redox reactions where δ in the Ag/Sr_0.85_Ag_0.15_FeO_3−δ_ electrode adsorbed the electrolyte ions^26,52^. The existence of Fe^3+^/Fe^4+^ and oxygen vacancies in this composite is confirmed by the above XPS and Mössbauer measurements. The Ag/Ag_2_O redox reactions can be attributed to the Ag particles which are confirmed by above XRD, TEM, and XPS measurements. Based on XPS spectra of Ag and Fe as well as Mössbauer spectrum, the conversion of Ag and Fe state in the Ag/Sr_0.85_Ag_0.15_FeO_3−δ_ composite can be explained by the following chemical reactions^12,22^:

7 $$2Ag+2O{H^ - } \leftrightarrow A{g_2}O+{H_2}O+2{e^ - }$$

8 $$S{r_{0.85}}A{g_{0.15}}\left[ {Fe_{{0.85 - 2\delta }}^{{4+}};Fe_{{0.15+2\delta }}^{{3+}}} \right]O_{{3 - \delta }}^{{2 - }}+2\delta O{H^ - } \leftrightarrow S{r_{0.85}}A{g_{0.15}}\left[ {Fe_{{2\delta }}^{{4+}}} \right]+\delta {H_2}O+2\delta {e^ - }$$

where OH^−^ represents the hydroxyl in the alkaline electrolyte (6 M KOH) and δ represents oxygen vacancies. The charge storage in these chemical reactions may be described through the generation of O^2−^ and H_2_O during the charge which result from the absorption of OH^−^. Subsequently O^2−^ diffuse to fill the oxygen vacancies sites and Fe^3+^ can be oxidized to Fe^4+^^9^. As the scan rate increases, it is noticed that the small redox peaks of Ag/Ag_2_O are weakening due to the insufficient reaction of electron neutralization^12^. The oxidation and reduction peaks of Fe were also shifted to higher potential and lower potential, respectively. This behavior can be due to the presence of fast electronic at a high scanning rate^1^. From the above observation, it can con be concluded that the presence of Ag nanoparticles in present composite leads to the acceleration of charge transfer and contributes in pseudocapacitance with a small amount^12^. To further clarify the electrochemical behaviors of the present composite, we use the power law relationship with the scan rate, as given in the following equation^22^.

9 $$i=a{v^b}$$

where i is the anodic or cathodic peak current (mA), a and b are adjustable parameters, and ν is the scan rate (mV s^− 1^). Figure 11b displays the plot of log (i) against log (ν) for the present composite. The value of b of this composite can be obtained from the slope of the linear plot of log (i) against log (ν). The b values are 0.74 and 0.73 for anodic or cathodic peak, respectively. These values indicate the charge storage of this composite which was obtained from a mixture process involving capacitive and linear diffusion^12,22^.

**Fig. 12:**
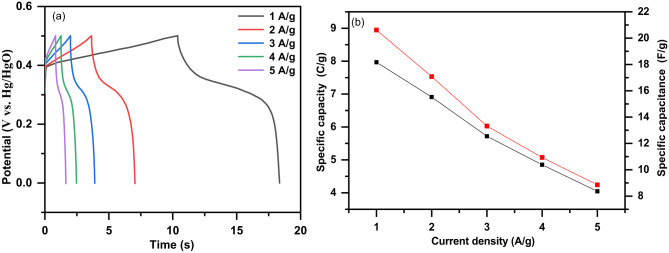
**a** The galvanostatic charge-discharge (GCD) curves of the Ag/Sr_0.85_Ag_0.15_FeO_3−δ_ composite at different current densities of 3, 4, and 5 A/g. **b** The specific capacity (C) and specific capacitance (C_s_) of the present composite, respectively.

**Fig. 13:**
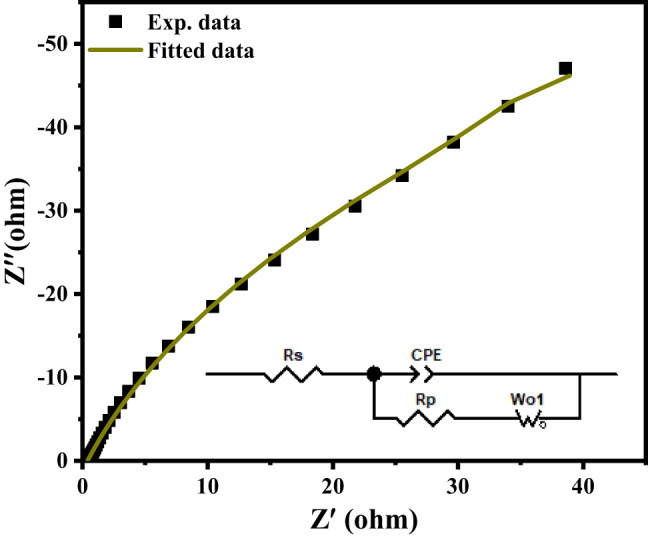
The Nyquist plot of the Ag/Sr_0.85_Ag_0.15_FeO_3−δ_ composite and the inset shows the equivalent circuit of the Ag/Sr_0.85_Ag_0.15_FeO_3−δ_ electrode.

Figure 12a displays the GCD curves of the Ag/Sr_0.85_Ag_0.15_FeO_3−δ_ electrode at different current densities of 1, 2, 3, 4, and 5 A/g, respectively. It can be noted that these curves show a rapid potential drop on the left side and the potential decay on the right side. This observation confirmed the existence of pseudocapacitive behavior of the Ag/Sr_0.85_Ag_0.15_FeO_3−δ_ electrode, which is in good agreement with redox peaks observed in CV curves^22^. Additionally, these curves exhibit discharge plateau at a high current density of 5 A/g. which indicates the good electrochemical performance of the present composite^12^. Figure 12b displays the plot of the specific capacity (C) and specific capacitance (C_s_) of the Ag/Sr_0.85_Ag_0.15_FeO_3−δ_ electrode as a function of current density. It is noticed that the C and C_s_ values of this composite decreases with increasing the current density which means the presence of good supercapacitors performance. This result can be due to the internal resistance consumption and increasing polarization effect^24^. Figure 13 shows the Nyquist plot obtained from the EIS data of the Ag/Sr_0.85_Ag_0.15_FeO_3−δ_ electrode over the frequency range of 100 kHz to 100 MHz. It can be noted that the Nyquist plot displays a curved line at high frequency and nearly straight line at low frequency region. The Nyquist plot was analyzed by Zview™ software based on the equivalent circuit as shown in inset of Fig. 13. In the equivalent circuit, Rs is series resistance, CPE is the constant phase element, Rp is the charge transfer resistance and Wo is Warburg resistance^53^. The Nyquist fitting results such as R_S,_ R_p,_ and Warburg diffusion resistance (W_0_) values of Ag/Sr_0.85_Ag_0.15_FeO_3−δ_ electrode are equal to 0.40, 121.5 and 529 Ω, respectively, and the capacitance (CPE_T_), the constant phase element exponent (CPE_P_), the diffusion time constant (W_T_) and a fractional exponent (W_p_) are equal to 0.015, 0.787, 20.41, and 0.921 respectively. The smaller values of R_S_ and R_p_ of the Ag/Sr_0.85_Ag_0.15_FeO_3−δ_ electrode indicate the lower activation energy for the electron transfer reaction and the fast of the oxygen reduction reaction, respectively^40^. In addition, the smaller values of R_S_ and R_p_ in the present composite are due to the high content of oxygen vacancies (δ) which increase the electrical conductivity and the dynamics of the charge transfer^24^. The existence of oxygen vacancy sites in the structure of this composite leads to the increase pathways for the diffusion of the electrolyte ions^9^. The high value of W_0_ indicates the less contact between the active material and Ni foam^53^.

### Antibacterial properties of Ag/Sr_0.85_Ag_0.15_FeO_3−δ_ composite

**Fig. 14:**
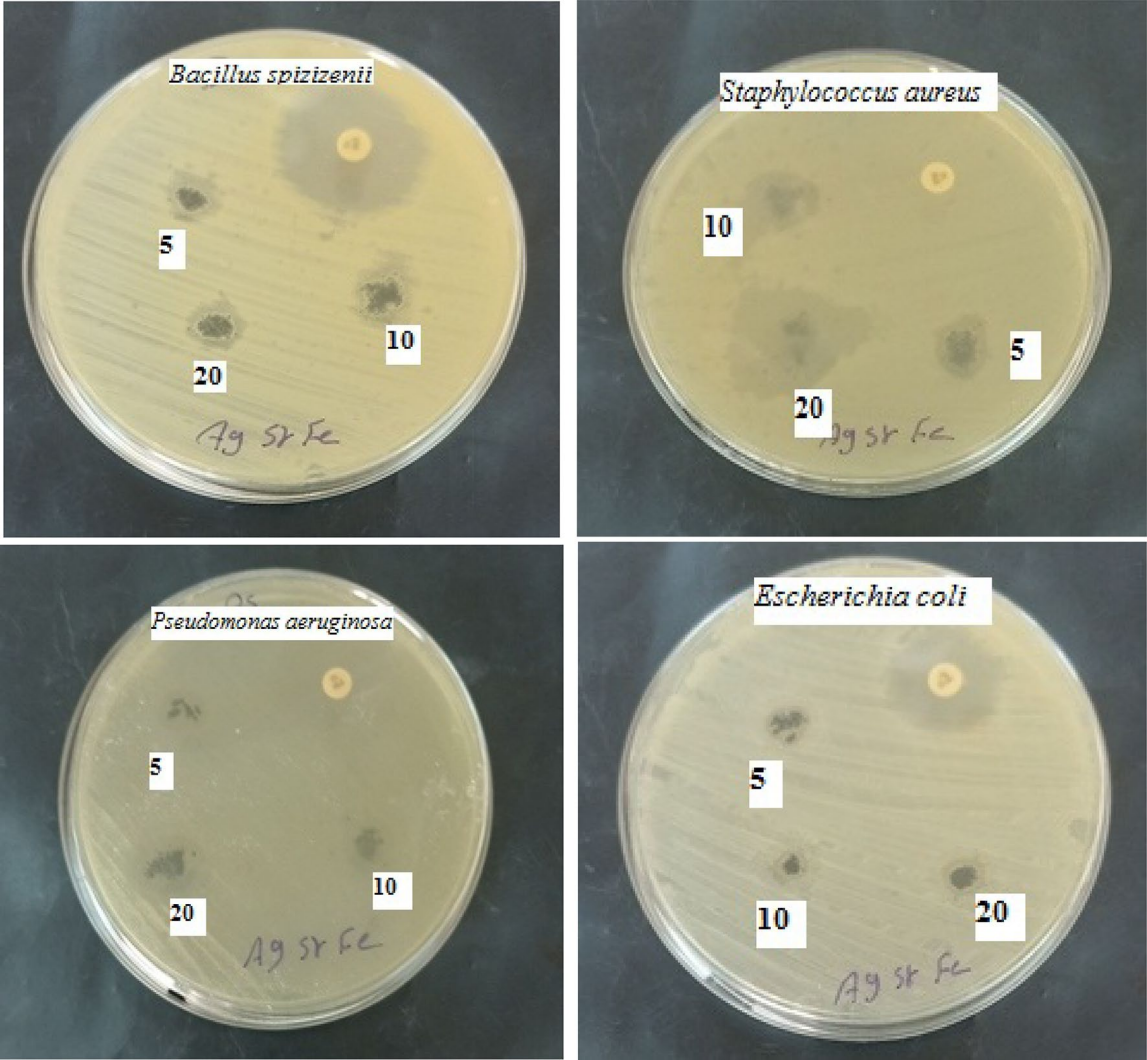
The antimicrobial activity (*Bacillus spizizenii*,* Staphylococcus aureus*,* Pseudomonas aeruginosa and Escherichia coli)* of the Ag/Sr_0.85_Ag_0.15_FeO_3−δ_ composite at concentrations of 5, 10, and 20 mg.

**Fig. 15:**
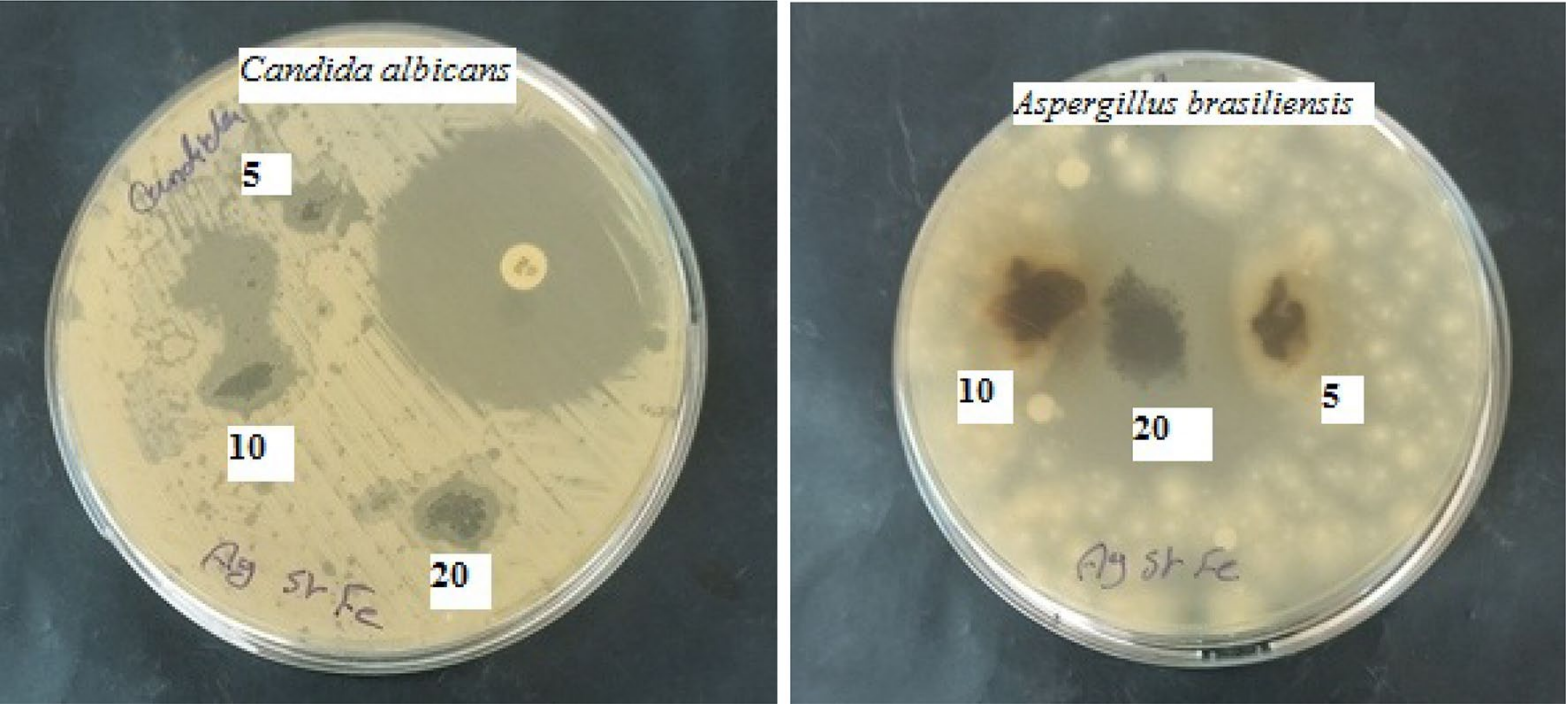
The antifungal activity of Ag/Sr_0.85_Ag_0.15_FeO_3−δ_ composite *(Candida albicans* (ATCC 10231)) and *Aspergillus brasiliensis (ATCC 16404))* at concentrations of 5, 10, and 20 mg.

Figure 14 shows the antibacterial activity of Ag/Sr_0.85_Ag_0.15_FeO_3−δ_ composite against Gram-positive (*Bacillus spizizenii and Staphylococcus aureus*) and Gram-negative (*Pseudomonas aeruginosa and Escherichia coli*) bacteria at concentrations of 5, 10, and 20 mg. The antibacterial activity screening method in the present composite is the inhibition zone. The inhibition zone diameters of the investigated composite against gram-positive and gram-negative bacteria as a function of concentrations are tabulated in Table 3. It is noted that the inhibition zone diameters for the gram positive and gram-negative bacteria increase with increasing the concentration of the composite sample from 5 to 20 mg. *Staphylococcus aureus and Pseudomonas aeruginosa* bacteria exhibited resistance to the current antibiotic (Imipenem) but the present composite exhibited antibacterial activity for these types of bacteria as shown in Fig. 14; Table 3. Moreover, the inhibition zone of gram-positive (*Staphylococcus aureus*) is larger than that of gram-negative bacteria (*Escherichia coli*) while the inhibition zone of gram-negative *(Pseudomonas aeruginosa)* is larger than that of gram-positive bacteria (*Bacillus spizizenii)* at all concentrations. This result can be attributed to the difference in both the morphology and the wall of the cells of the bacteria^14,16^. The damage and cell death of both gram positive and gram-negative bacteria could be due to the presence of small spherical particle size of Ag which can easily reach the nuclear content of the bacteria^3,13^. Additionally, the toxic effect of the present composite can be due to the presence of reactive oxygen species (ROS) and the liberation of Sr^2+^ ions which make the environment around the bacteria unhealthy^3,16^. Figure 15. shows the antifungal activity of Ag/Sr_0.85_Ag_0.15_FeO_3−δ_ composite against unicellular *(Candida albicans* (ATCC 10231)) and filamentous fungi *(Aspergillus brasiliensis (ATCC 16404))* at concentrations of 5, 10, and 20 mg. The inhibition zone diameters of the investigated composite against unicellular and filamentous fungi at different concentrations are tabulated in Table 3. It is noted that the diameter of the inhibition zone for *Candida albicans* fungi increases with increasing the concentration of the composite while *Aspergillus terreus* fungi exhibited antifungal activity at high concentration only (20 mg). This observation can be attributed to the small spherical particle size of Ag in the present composite which disrupts fungal physiology^3,15^.

## Conclusion

Ag/Sr_0.85_Ag_0.15_FeO_3−δ_ composite was prepared by the sol-gel method. From XRD, TEM and SAED analysis, we concluded that there are orthorhombic (space group Pnma) structure of Sr_0.85_Ag_0.15_FeO_3−δ_ perovskite and cubic (space group Fm3m) structure of Ag metallic in this composite. The average particle size of Ag in this composite is 7.72 nm which was evaluated by TEM. According to XPS and Mössbauer spectral analysis, we concluded that there are oxygen vacancies (δ), Fe^3+,^ and Fe^4+^ ions in this composite. Optical results of this composite revealed that the value of E_g_ is 3.53 eV and lies in the UV region. M-H hysteresis loop of this composite showed the exchange bias effect at room temperature. Electrochemical results indicated that the Ag/Sr_0.85_Ag_0.15_FeO_3−δ_ composite can be a good candidate of electrode material for energy storage applications. The antibacterial tests of this composite showed strong activity to fight some types of gram-positive (*Staphylococcus aureus* (ATCC 6538)) and gram-negative (*Pseudomonas aeruginosa* (ATCC 9027)) bacteria that resist the current antibiotic (Imipenem). Finally, these findings indicate that this composite can be used as a promising material for magnetic, biomedical, and energy storage applications.

## Data Availability

All the data are available within this paper.
